# Type 2 diabetes among people with selected citizenships in Germany: risk, healthcare, complications

**DOI:** 10.25646/12159

**Published:** 2024-06-26

**Authors:** Maike Buchmann, Carmen Koschollek, Yong Du, Elvira Mauz, Laura Krause, Laura Neuperdt, Oktay Tuncer, Jens Baumert, Christa Scheidt-Nave, Christin Heidemann

**Affiliations:** Robert Koch Institute, Department of Epidemiology and Health Monitoring, Berlin, Germany

**Keywords:** Migration, Discrimination, Health inequality, Type 2 diabetes, Germany, Depression, Communication barriers

## Abstract

**Background:**

Migration-related factors, such as language barriers, can be relevant to the risk, healthcare and complications of type 2 diabetes in people with a history of migration. Diabetes-related data from people with selected citizenships were analysed on the basis of the nationwide survey German Health Update: Fokus (GEDA Fokus).

**Methods:**

The diabetes risk of persons without diabetes (n = 4,698, 18 – 79 years), key figures on healthcare and secondary diseases of persons with type 2 diabetes (n = 326, 45 – 79 years) and on concomitant diseases (n = 326 with type 2 diabetes compared to n = 2,018 without diabetes, 45 – 79 years) were stratified according to sociodemographic and migration-related characteristics.

**Results:**

Better German language proficiency is associated with a lower risk of diabetes. Diabetes-related organ complications are observed more frequently in persons who report experiences of discrimination in the health or care sector. Both persons with and without diabetes are more likely to have depressive symptoms when they reported experiences of discrimination. A stronger sense of belonging to the society in Germany is associated with reporting depressive symptoms less often in people without diabetes, but not in people with type 2 diabetes.

**Conclusions:**

The differences according to migration-related characteristics indicate a need for improvement in the prevention and care of type 2 diabetes. Migration-sensitive indicators should be integrated into the surveillance of diabetes.

## 1. Introduction

People with a history of migration, i.e. people who themselves or whose parents have immigrated to Germany [1], make up around a quarter of the population in Germany [2]. There is numerous evidence that the relationship between migration and health is complex, with knowledge of the majority language and social situation playing a role, among others [3]. With regard to the risk of chronic diseases, there are different approaches to explain which migration-related characteristics are relevant [3, 4]. Depending on the course of the migration process and the living conditions in the destination country, pre-existing health risks may be further exacerbated. In Germany, people with a history of migration are often socioeconomically disadvantaged [5] and affected by (racial) discrimination [6]. This can result in chronic stress and unfavorable nutrition and physical activity habits, e.g. due to stressful working and living conditions, which increase the risk of non-communicable diseases [6, 7]. Health inequalities are further exacerbated if services in the healthcare system are not sufficiently accessible and tailored to the needs of people with a history of migration. Experiences of discrimination can have a negative impact on mental and physical health [8, 9]. They can also lead to hampered or delayed access to medical treatment [6, 10]. People with lower levels of German language proficiency can also encounter language barriers in the healthcare system [11].

In order to collect comprehensive data on the health situation of people with a history of migration, the health interview survey ‘German Health Update: Fokus (GEDA Fokus)’ was conducted by the Robert Koch Institute (RKI) with a sample of people with selected citizenships in order to depict some of the major migration groups in Germany. Previously published analyses based on GEDA Fokus data show that experiences of discrimination in everyday life as well as in the health or care sector, lower German language proficiency or a lower sense of belonging to the society in Germany can be associated with health risks and unfavorable health conditions [1, 12]. For example, a higher proportion of smokers was observed among people with self-reported experiences of discrimination in everyday life compared to those who rarely or never reported discrimination [1]. In addition, both experiences of discrimination and a lower sense of belonging to the society in Germany were associated with poorer subjective health, depressive symptoms and the presence of a chronic disease or long-term health problem [1, 12]. Furthermore, lower German language proficiency was associated with utilising general practicioners’ services less often [1]. GEDA Fokus also collected data on risk factors and healthcare-related aspects of diabetes mellitus, for which the aforementioned migration-related characteristics might also be of importance.

Diabetes mellitus is a chronic disorder of the blood sugar metabolism that is associated with elevated blood sugar levels. Lifelong treatment is usually necessary to avoid acute metabolic imbalances and serious secondary diseases such as kidney failure, blindness, diabetic foot ulcers and lower limb amputations. In order to achieve a stable metabolic situation, a favorable course of the disease and a good quality of life, it is important for people with diabetes to receive continuous and trustworthy treatment. This requires regular medical examinations and good self-management (e.g. by self-monitoring blood glucose) [13]. In contrast to autoimmune type 1 diabetes, in which the body’s own vital insulin can no longer be produced and must be replaced, in type 2 diabetes the transport of blood glucose into the cells is restricted due to a reduced insulin effect (insulin resistance). In favorable cases, a change in diet and exercise habits is sufficient to counteract insulin resistance. Options for drug-based blood glucose-lowering treatment are constantly evolving and the recommendations of the National Treatment Guideline for type 2 diabetes are continuously adapted [13]. The known risk factors for type 2 diabetes include older age, genetic factors and, above all, controllable risk factors that are also relevant for the development of other common non-communicable diseases such as cancer and cardiovascular diseases. These include unfavorable nutrition habits, physical inactivity, obesity and smoking as well as living and working conditions that favor these behaviours and environmental factors (e.g. air pollution) [14–17]. In addition, adults with type 2 diabetes have an increased risk for concomitant diseases such as high blood pressure and lipometabolic disorders as well as depressive symptoms compared to people without diabetes [18, 19] which further increase the risk of diabetes-related secondary diseases. The incidence of type 2 diabetes has risen sharply worldwide in recent years. Effective prevention measures are urgently needed at a societal level [20, 21].

To support evidence-based policy decisions to prevent diabetes and improve the health situation of people with diabetes in Germany, the diabetes surveillance project was established at the RKI (www.diabsurv.rki.de) [22]. It summarises data on the risk, healthcare, concomitant and secondary diseases and disease burden of diabetes in Germany [23]. The indicators are differentiated by sex, age and, where possible, by region and individual level of education or regional socioeconomic deprivation. Due to health inequalities faced by people with a history of migration [1, 12] the surveillance (i.e. continuous monitoring of the health situation of the population) of diabetes in Germany should consider not only individual, social and environmental determinants of health but also migration-related characteristics. Overall, the data available on diabetes in people with a history of migration in Germany is still insufficient [24–26]. Based on GEDA Fokus data, the study at hand therefore aims to present results on type 2 diabetes risk, healthcare and secondary and concomitant diseases associated with type 2 diabetes in people with Croatian, Italian, Polish, Syrian or Turkish citizenship living in Germany. In particular, it will be analysed whether there are differences according to the aforementioned migration-related characteristics, i.e. experiences of discrimination in everyday life or in the health or care sector, sense of belonging to the society in Germany and German language proficiency, in order to obtain information on potential prevention and healthcare needs with regard to diabetes.

## 2. Method

### 2.1 Study design and study population

GEDA Fokus (see Infobox) is a multilingual, multimodal health interview survey among people with Croatian, Italian, Polish, Syrian or Turkish citizenship aged 18 to 79 living in Germany, which was conducted nationwide from November 2021 to May 2022.

Sample persons were randomly drawn from the residents’ registries of 99 cities and municipalities according to their citizenship (1st, 2nd or 3rd citizenship, including dual citizenship). The selection of citizenships was based on model calculations [27] and the citizenships included represent some of the largest migration groups in Germany. The selected sample persons were offered to take part in the survey in a sequential process, first via a web-based questionnaire for which login data were provided in an initial invitation letter send by mail and in a second step via a paper-based questionnaire, which was sent to them with a reminder letter. In a third step, all those who had neither participated nor declined to participate received a second reminder letter; in larger cities, additionally a home visit was announced to conduct a personal or telephone interview, which took place in the fourth step. All study materials and questionnaires were bilingual (e.g. German-Italian) and some of the interviewers who conducted the home visits were bilingual as well. Further details on the study design can be found elsewhere [27].

A total of 6,038 people (2,983 women, 3,055 men; mean age: 41.6 years, 95 % confidence interval (95 % CI): 41.2 – 42.0) took part in GEDA Fokus. The response rate according to the standards of the American Association for Public Opinion Research (AAPOR) was 18.4 % (response rate 1) [28].

The absolute 5-year risk of developing type 2 diabetes was calculated in a subsample of 4,698 participants aged 18 to 79 years (2,349 women, 2,349 men) who negated the question about ever having been diagnosed with diabetes by a doctor and for whom complete information was available to calculate the diabetes risk. The analyses of healthcare and secondary diseases of type 2 diabetes are based on a sub-sample of 326 participants aged 45 to 79 years (140 women, 186 men) who are assumed to have type 2 diabetes (diagnosed by a physician) on the basis of self-reported data. For this purpose, people who are assumed to have type 1 diabetes (n = 23) or gestational diabetes (n = 27) were initially excluded from the group of people who confirmed a physician’s diagnosis of diabetes (n = 441) [29]. In addition, people under the age of 45 were excluded (n = 65), as type 2 diabetes is more common from middle age onwards. The analyses on concomitant diseases differentiated between persons with type 2 diabetes aged 45 to 79 years and a group of 2,018 participants without diabetes in the same age group (1,032 women, 986 men) to allow for comparisons.

**German Health Update: Fokus (GEDA Fokus)**

**Data holder:** Robert Koch Institute

**Objectives:** To describe the health situation of people with selected citizenships utilising a set of core indicators [30]. Differentiated analyses on health status, health behaviour and the utilisation of health services according to sociodemographic, socioeconomic and migration-related characteristics, e.g. duration of residence, German language proficiency or self-reported experiences of discrimination

**Study design:** Cross-sectional survey

**Population:** Adults aged between 18 and 79 living in Germany with Croatian, Italian, Polish, Syrian or Turkish citizenship (including dual citizenship). The selection of citizenships is based on model calculations considering the size of the citizenship groups and the dynamics (in-and outward migration) using data from the foreigner statistics 2015 – 2017 provided by the Federal Office for Statistics [27]

**Sampling:** Two-stage sampling procedure: (1) 120 primary sampling units (sample points) randomly selected nationwide in 99 cities and municipalities, (2) random sampling of persons aged 18 to 79 years from the respective residents’ registries according to the characteristic of 1st, 2nd or 3rd citizenship (including dual citizenship)

**Survey method:** Web- or paper-based questionnaire, telephone or face-to-face interview, six study languages (Arabic, Croatian, German, Italian, Polish and Turkish)

**Sample size:** 6,038 persons (2,983 women, 3,055 men)

**Data collection period:** November 2021 – May 2022

**Data protection:** The participants were informed about the objectives and content of the study and about data protection regulations and gave their informed consent to participate in the study

More information at https://doi.org/10.2196/43503

### 2.2 Survey content and instruments

#### Type 2 diabetes risk

The German Diabetes Risk Score (GDRS), which estimates the 5-year probability of developing type 2 diabetes, was used to assess type 2 diabetes risk in 18- to 79-year-olds without prior diagnosis of diabetes. The updated and validated simplified version of the test was used, which includes age (10 categories), waist circumference (11 categories), height (7 categories), physical activity (2 categories), smoking (5 categories), high blood pressure (yes/no), diabetes in biological parents (3 categories) and siblings (2 categories), consumption of red meat (6 categories), consumption of whole grain products (6 categories) and coffee consumption (3 categories) as components of the score to be calculated [31, 32]. As information on family history of diabetes was only available for those individuals who reported a medical diagnosis of diabetes, the prevalences of diabetes observed in other population-based studies in one parent (24.0 %), both parents (2.0 %) or at least one sibling (5.0 %) were used as constants in accordance with the procedure in a previous analysis [33]. Waist circumference was determined using a sex-specific regression equation from self-reported height, weight and age [34]. Based on published algorithms, points were assigned to each component according to its severity, the sum score of which was then used to calculate the absolute risk [31]. For the current analysis, the diabetes risk was categorised as ‘low risk’ (< 2 %), ‘still low risk’ (2 to < 5 %), ‘elevated risk’ (5 to < 10 %) and ‘high to very high risk’ (≥ 10 %) based on the classification used for risk communication of GDRS results [35].

#### Healthcare for type 2 diabetes

Aspects of healthcare are analysed for 45- to 79-year-olds with type 2 diabetes. The following answer options were available for the question ‘How are you currently being treated?’: ‘With diet’, ‘With tablets’, ‘With insulin’, ‘With other blood glucose-lowering medication that is injected (except insulin)’. In addition, respondents were asked whether they check their blood glucose levels themselves (yes/no). Furthermore, respondents were asked to state when their blood glucose levels were last measured by a healthcare professional (possible answers: ‘Within the last 4 weeks’, ‘Within the last 2 to 12 months’, ‘1 to less than 3 years ago’, ‘3 to less than 5 years ago’, ‘5 years ago or more’, ‘Never’). The response categories were combined to determine whether a check had been carried out in the last 12 months (yes/no).

#### Complications of type 2 diabetes

In 45- to 79-year-old people with type 2 diabetes, diabetes-related secondary diseases or complications were depicted. These were queried using a list of ‘complications caused by diabetes’. The following complications were included in this study: diabetes-related kidney disease, diabetes-related eye disease, diabetes-related nerve problems, diabetic foot and amputations. A distinction was made between affirming at least one of the diabetes-related complications mentioned here and negating all of the complications.

#### Concomitant diseases in relation to type 2 diabetes

For the analysis of concomitant diseases (comorbidities), in addition to the 45- to 79-year-olds with type 2 diabetes, people in the same age range without diabetes were also considered. The presence of depressive symptoms was recorded using the validated Patient Health Questionnaire-8 (PHQ- 8) [36]. A sum score of at least 10 on the scale (score range: 0 – 24) was considered to indicate the presence of depressive symptoms according to international standards [36]. Cardiovascular comorbidity (yes/no) was assumed if at least one of a total of four questions about the lifetime medical diagnosis of (1) a circulatory disorder of the heart, constriction of the coronary arteries or angina pectoris, (2) a heart attack (myocardial infarction), (3) cardiac insufficiency or heart failure or (4) a stroke was affirmed. The presence of high blood pressure was determined by asking for a lifetime medical diagnosis of high blood pressure or hypertension (yes/no).

### 2.3 Sociodemographic and migration-related characteristics

Sex was differentiated into female and male based on the sex entered on the birth certificate. Age was categorized into groups of 18- to 44-year-olds, 45- to 64-year-olds and 65- to 79-year-olds. Education groups were classified using information on highest school and vocational training qualifications and grouped according to the Comparative Analysis of Social Mobility in Industrial Nations (CASMIN) classification [37]. For the analyses, the medium and high education groups were combined and compared with the low group due to small number of cases.

The respondents’ German language proficiency was surveyed in two stages. Firstly, they were asked about their native language (answer options: ‘German’ and ‘another language’). Those who did not state German as their native language were asked to rate their German language proficiency (answer options: ‘very good’, ‘good’, ‘moderate’, poor’, ‘very poor’). In the analyses, the responses to both questions were combined and categorized as ‘native language/very good/good’ and ‘moderate/poor/very poor’ due to small number of cases.

The frequency of subjectively perceived experiences of discrimination in everyday life was surveyed using five questions. These asked how often 1) respondents were treated with less politeness or respect than other people, 2) they received poorer service than other people, 3) someone behaved as if he or she does not take them seriously, 4) someone behaved as if he or she was afraid of them or 5) they were threatened or harassed [1, 38]. In the analyses, a distinction was made between people who answered ‘very often’, ‘often’ or ‘sometimes’ to at least one of the five questions and people who answered ‘rarely’ or ‘never’ to all questions [1]. Furthermore, using the same response options, the frequency of self-reported experiences of discrimination in the health or care sector was investigated. Here, the categories ‘very often/often/sometimes’ and ‘rarely/never’ were combined for the analyses as well.

The possible answers to the question ‘How much do you feel you belong to the society in Germany?’ were grouped into the categories ‘partly/barely/not at all’ and ‘very strongly/ strongly’ due to small number of cases [38].

### 2.4 Statistical methods

All indicators are presented as proportions (prevalence) with 95 % confidence intervals (CI) and stratified according to the sociodemographic and migration-related characteristics described above. A statistically significant group difference was assumed for a p-value of less than 0.05 determined in Rao-Scott chi-square test. Linear and logistic regression analyses, respectively, were calculated to determine if associations found in bivariate analyses could be confirmed between diabetes-relevant characteristics (i.e. type 2 diabetes risk, healthcare indicators or secondary and concomitant diseases) and migration-related characteristics while controlling (adjusting) for sex (women, men), age (18 – 44, 45 – 64, 65 – 79 years), education (low vs. medium and high according to CASMIN [37]) and citizenship (according to residents’ registries).

With regard to comorbidities, Prevalence ratios (PR, with 95 % CI) were calculated on the basis of Poisson regressions, additionally. These describe the ratio of the prevalence of these comorbidities in persons with type 2 diabetes compared to the prevalence in persons without prior diabetes diagnosis.

In order to account for deviations between the sample and the population with the selected citizenships in the distribution of sex, age, education (according to the International Standard Classification of Education, ISCED), region and duration of residence [39] a weighting factor was used in the analyses. This weighting is based on data from the 2018 microcensus for the corresponding citizenships (including dual citizenship) [39]. The different probabilities for the selection and clustering of participants in the cities and municipalities associated with the study design were considered by applying survey procedures for complex samples. The analyses were conducted using Stata 17.0 and SAS 15.2 [40, 41].

## 3. Results

### 3.1 Characteristics of the samples

The three sub-samples analysed are presented according to sociodemographic and migration-related characteristics in Table 1. The sub-sample for which the type 2 diabetes risk was calculated comprises persons without a diabetes diagnosis in the age range 18 – 79 years (mean age: 40.9 years, 95 % CI: 40.0 – 41.8, proportion of persons born in Germany: 22.0 %, 95 % CI: 19.4 – 24.9, mean duration of residence for persons not born in Germany: 22.3 years, 95 % CI: 20.7 – 24.0). The sub-sample, for which key figures on healthcare, secondary and concomitant diseases are shown, includes persons with type 2 diabetes in the age range 45 – 79 years (mean age: 62.7 years, 95 % CI: 61.0 – 64.3, proportion of persons born in Germany: 3.0 %, 95 % CI: 1.5 – 5.9, mean duration of residence for persons not born in Germany: 39.3 years, 95 % CI: 37.5 – 41.1). The sub-sample used for comparison with regard to depressive symptoms, cardiovascular disease and high blood pressure comprises of persons without a diabetes diagnosis in the age range 45 – 79 years (mean age: 56.3 years, 95 % CI: 55.6 – 57.0, proportion of persons born in Germany: 7.4 %, 95 % CI: 5.8 – 9.5, mean duration of residence for persons not born in Germany: 33.3 years, 95 % CI: 31.9 – 34.7). A detailed description of the overall population of GEDA Fokus can be found elsewhere [12, 27].

### 3.2 Type 2 diabetes risk

The geometric mean of the 5-year risk of developing type 2 diabetes according to GDRS is 0.9 % for 18- to 79-year-olds without a previous diabetes diagnosis, i.e. on average around one person in 100 will be diagnosed with type 2 diabetes within the next 5 years (women 0.6 %, men 1.1 %). A low risk is present in 71.9 % (95 % CI: 69.1 – 74.4). In 14.0 % (95 % CI: 12.3 – 15.8), the risk can be categorised as ‘still low’ according to the classification for risk communication of GDRS results, and in 7.4 % (95 % CI: 6.1 – 8.9) as ‘elevated’. Overall, 6.8 % (95 % CI: 5.6 – 8.1) have a high to very high risk of developing type 2 diabetes [35].

A lower type 2 diabetes risk is more frequent in women than in men. The proportion of persons with an elevated risk and a high to very high risk increases significantly with age. There are also differences according to education, with a proportion as twice as high among persons with a high to very high risk in the low compared to the medium and high education group (Annex Table 1).

**Figure 1:**
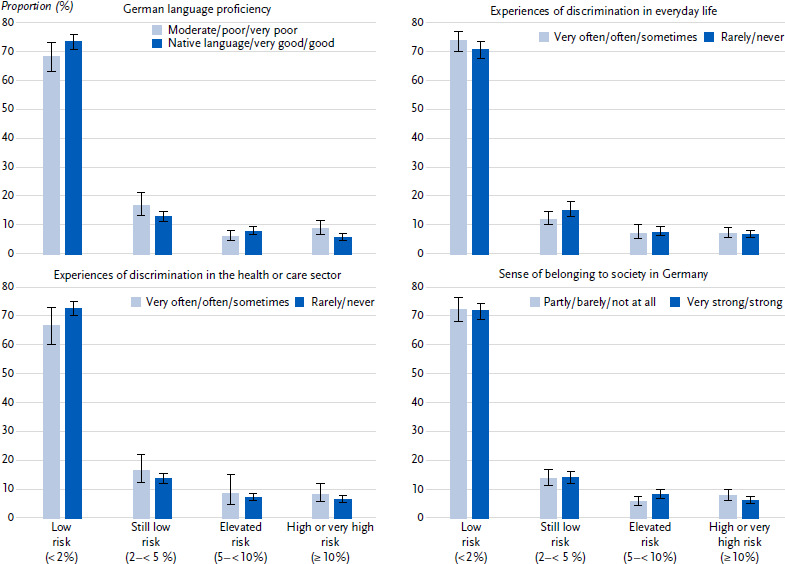
Type 2 diabetes risk (proportions with 95 % confidence intervals) among people with selected citizenships in Germany (18 – 79 years) without diabetes according to German language proficiency, experiences of discrimination in everyday life and in the health or care sector and sense of belonging to the society in Germany (n = 2,349 women, n = 2,349 men). Source: GEDA Fokus Missing values: n = 35 for German language proficiency, n = 3 for experiences of discrimination in everyday life, n = 20 for experiences of discrimination in the health or care sector, n = 44 for sense of belonging to the society in Germany

The proportion of persons with a high to very high risk is lower among people who report to be native-speaker or have very good or good German language proficiency (5.7 %; 95 % CI: 4.6 – 7.2) than among persons with less good German language proficiency (8.8 %; 95 % CI: 6.5 – 11.9 (Figure 1), p = 0.019). The association between German language proficiency and type 2 diabetes risk can also be observed in the multivariable analysis (p = 0.004). No differences in the distribution of diabetes risk were observed when differentiating according to subjectively perceived experiences of discrimination in everyday life and in the health or care sector and with regards to the sense of belonging to the society in Germany (Figure 1).

### 3.3 Healthcare for type 2 diabetes

8.8 % (95 % CI: 4.1 – 17.9) receive no blood glucose-lowering medication (antidiabetics), while 91.2 % (95 % CI: 82.1 – 95.9) are treated pharmacologically, including 28.3 % (95 % CI: 20.0 – 38.4) with insulin (alone or in combination with other antidiabetics) and 62.9 % (95 % CI: 53.1 – 71.8) with tablets or other injectable medications (except insulin). While there are no sex-related differences, the proportion of 45- to 64-year-olds who do not receive antidiabetic medication is higher than among 65- to 79-year-olds. Persons in the low compared to those in the middle and high education group receive insulin therapy more frequently (exclusively or in combination with other antidiabetics). There are no differences in the type of medication according to German language proficiency, self-reported experiences of discrimination in everyday life or in the health or care sector, or according to the sense of belonging to the society in Germany (Annex Table 2).

With regard to blood glucose self-monitoring, more than half of the persons with type 2 diabetes aged 45 to 79 stated that they measured their blood glucose themselves (57.4 %, 95 % CI: 47.9 – 66.4). A total of 93.0 % (95 % CI: 86.8 – 96.4) stated that their blood glucose had been measured by a healthcare professional within the last twelve months. No sex-related or education-related differences were observed with regard to self-monitoring of blood glucose and measurement by a healthcare professional. However, people aged between 65 and 79 were slightly more likely than 45- to 64-year-olds to state that their blood glucose had been checked by a healthcare professional within the last year (p = 0.049) (Annex Table 3).

Both, self-monitoring of blood glucose and measurement by a healthcare professional within the last year, do not vary according to German language proficiency, self-reported experiences of discrimination in everyday life or in the health or care sector or the sense of belonging to the society in Germany (Annex Table 3).

### 3.4 Complications of type 2 diabetes

Overall, 32.6 % (95 % CI: 22.2 – 45.0) of respondents aged 45 to 79 years with type 2 diabetes reported the presence of at least one of the diabetes-related complications analysed, i.e. kidney, eye or nerve disease, diabetic foot syndrome or amputation.

There were no differences in the prevalence of diabetes-related complications according to sex, age or education (Annex Table 4).

Persons who stated that they sometimes, often or very often experienced discrimination in the health or care sector were more likely to have at least one diabetes-related complication (60.3 %, 95 % CI: 38.2 – 78.9) compared to persons who stated that they rarely or never had experienced discrimination in this context (26.2 %, 95 % CI: 16.6 – 38.6, (Annex Table 4), p = 0.001). Results of the multivariable analysis confirm the association between experiences of discrimination in the health or care sector and secondary diseases (p = 0.016). The prevalence of at least one of the selected complications did not differ according to German language proficiency, self-perceived experiences of discrimination in everyday life and sense of belonging (Annex Table 4).

### 3.5 Concomitant diseases with regard to diabetes

In the following, the proportions with depressive symptoms, cardiovascular disease and high blood pressure are described for people with type 2 diabetes, both overall and according to sociodemographic and migration-related characteristics. In comparison, the corresponding results for people without diabetes of the same age range are reported.

#### Depressive symptoms

Around one fifth of persons with type 2 diabetes has current depressive symptoms (20.5 %, 95 % CI: 14.2 – 28.8); this proportion does not differ significantly from the corresponding proportion of persons without a diabetes diagnosis (14.6 %, 95 % CI: 12.0 – 17.6, PR = 1.4, 95 % CI: 0.9 – 2.1).

When analysed according to sociodemographic characteristics, the proportion of women with type 2 diabetes with depressive symptoms is more than twice as high as that of men, while there are no age- or education-related differences. In people without diabetes, there are no differences according to sociodemographic characteristics (Annex Table 5).

**Figure 2:**
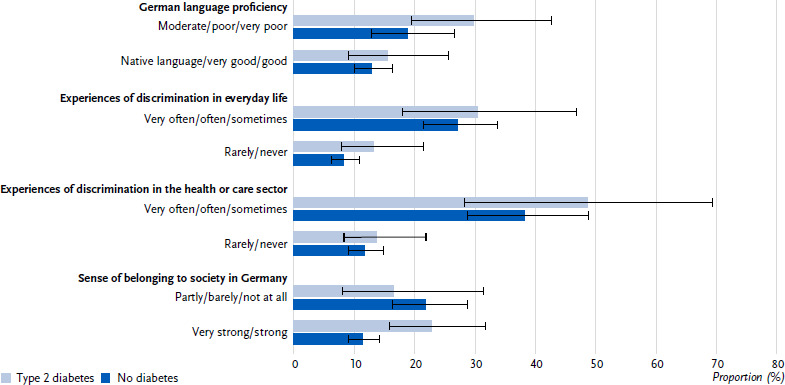
Depressive symptoms (proportions with 95 % confidence intervals) in people of with selected citizenships in Germany (45 – 79 years) with type 2 diabetes (n = 140 women, n = 186 men) compared to people without diabetes (n = 1,032 women, n = 986 men) according to German language proficiency, experiences of discrimination in everyday life and in the health or care sector and sense of belonging to the society in Germany. Source: GEDA Fokus Missing values: n = 12 for depressive symptoms in persons with type 2 diabetes, n = 55 for depressive symptoms in persons without diabetes

In bivariate analyses, in people with type 2 diabetes the proportion with depressive symptoms is higher among those with moderate to very poor German language levels (29.8 %, 95 % CI: 19.4 – 42.7) than among those with native, very good or good German language proficiency (15.6 %, 95 % CI: 9.0 – 25.7 (Figure 2), p = 0.042). This association could not be confirmed in the multivariable analysis (p = 0.235). Furthermore, bivariate analyses among persons with type 2 diabetes, revealed a higher proportion with depressive symptoms in those who sometimes to very often experience discrimination, both in everyday life (30.5 %, 95 % CI: 17.9 – 46.9) and in the health or care sector (48.6 %, 95 % CI: 28.2 – 69.4) compared to those who rarely or never report discrimination in these areas (13.2 %, 95 % CI: 7.8 – 21.6 (Figure 2), p = 0.023 and 13.7 %, 95 % CI: 8.2 – 22.0 (Figure 2), p < 0.001). These observed associations in people with type 2 diabetes also remain present in the multivariable analysis (p = 0.019 (discrimination in everyday life) and p = 0.004 (discrimination in the health or care sector)). Differences in the proportions with depressive symptoms are also evident for persons without diabetes according to experiences of discrimination in everyday life or in the health or care sector (Figure 2), which are also confirmed in the multivariable analyses (both p < 0.001). Among people without diabetes, but not among people with type 2 diabetes, a very strong or strong sense of belonging to the society in Germany is associated with less often reporting depressive symptoms compared to a lower sense of belonging to the society in Germany (Figure 2). The multivariable analyses also show an association in persons without diabetes (p < 0.001), and no association in persons with type 2 diabetes (p = 0.654).

#### Cardiovascular diseases

More than a third of persons with type 2 diabetes report a cardiovascular disease (38.7 %, 95 % CI: 30.2 – 48.0); this proportion is more than twice as high as that of persons without diabetes (14.7 %, 95 % CI: 11.9 – 18.0; PR: 2.6; 95 % CI: 1.9 – 3.6).

In persons with type 2 diabetes, the proportion with a cardiovascular disease is higher in the age group 65 and over than in the younger age group, while there are no differences by sex or education group. In persons without diabetes, there is also a higher proportion of cardiovascular disease in the older age group than in the younger age group and additionally a higher proportion in men than in women (Annex Table 5).

**Figure 3:**
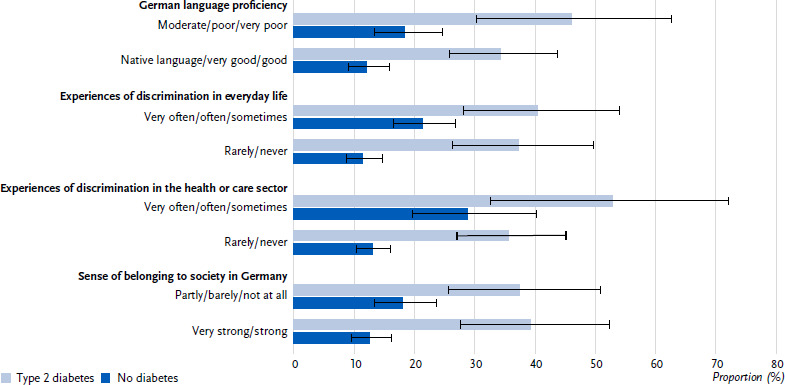
Cardiovascular diseases (proportions with 95 % confidence intervals) in people with selected citizenships in Germany (45 – 79 years) with type 2 diabetes (n = 140 women, n = 186 men) compared to people without diabetes (n = 1,032 women, n = 986 men) according to German language proficiency, experiences of discrimination in everyday life and in the health or care sector and sense of belonging to the society in Germany. Source: GEDA Fokus Missing values: n = 13 for cardiovascular disease in persons with type 2 diabetes, n = 72 for cardiovascular disease in persons without diabetes

Among people with type 2 diabetes, no significant differences were observed according to the migration-related characteristics analysed. In contrast, persons without diabetes with lower German language proficiency, with more frequent experiences of discrimination in everyday life or in the health or care sector and a lower sense of belonging to the society in Germany (in each case p < 0.05, except for sense of belonging p = 0.051) are more likely to report a cardiovascular disease (Figure 3). The differences observed in persons without diabetes are also confirmed in the multivariable analyses, except for the association between German language proficiency and cardiovascular disease (in each case p < 0.05, except for German language proficiency p = 0.193).

#### Hypertension

More than two thirds of persons with type 2 diabetes report high blood pressure (68.5 %, 95 % CI: 60.1 – 75.9); this proportion is more than twice as high as in persons without diabetes (31.2 %, 95 % CI: 27.8 – 34.7; PR: 2.2; 95 % CI: 1.9 – 2.6).

While there are no significant differences in the prevalence of hypertension among persons with diabetes with regard to the sociodemographic characteristics considered, among persons without diabetes hypertension is more frequently reported in the group aged 65 to 79 than in the younger age group (Annex Table 5).

**Figure 4:**
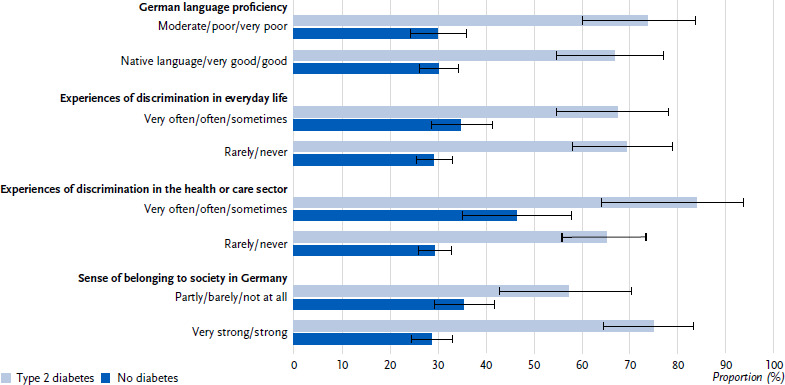
Hypertension (proportions with 95 % confidence intervals) in people of with selected citizenships in Germany (45 – 79 years) with type 2 diabetes (n = 140 women, n = 186 men) compared to people without diabetes (n = 1,032 women, n = 986 men) according to German language proficiency, experiences of discrimination in everyday life and in the health or care sector and sense of belonging to the society in Germany. Source: GEDA Fokus Missing values: n = 1 for hypertension in persons with type 2 diabetes, n = 35 for in persons without diabetes

The bivariate analyses show no associations between German language proficiency or experiences of discrimination in everyday life and high blood pressure, neither in people with type 2 diabetes nor in people without diabetes (Figure 4). Among persons with type 2 diabetes, in contrast to persons without diabetes, the proportion of high blood pressure is not lower among those who report no or rare experiences of discrimination in the health or care sector than among those who report more frequent experiences of discrimination (Figure 4). The multivariable analysis accordingly shows no association for persons with type 2 diabetes (p = 0.083), while an association is confirmed for persons without diabetes (p = 0.002). In persons with type 2 diabetes who report a stronger compared to a lower sense of belonging, the proportion with hypertension is higher (75.0 %, 95 % CI: 64.3 – 83.4 vs. 57.1 %, 95 % CI: 42.6 – 70.4 (Figure 4), p = 0.049), whereas this difference is not observed in persons without diabetes (Figure 4). The association between sense of belonging and hypertension observed among people with type 2 diabetes is not confirmed in the multivariable analysis (p = 0.107).

## 4. Discussion

The aim of the study was to identify characteristics of people with a history of migration that are associated with differences in the areas of type 2 diabetes risk as well as healthcare and secondary and concomitant diseases of type 2 diabetes in order to obtain information on potential prevention and care needs. Within the discussion of the results, key results that were found in the sample of people with selected citizenships are compared with results from previous analyses in samples of the general population with and without diabetes.

### 4.1 Discussion of the results

#### Type 2 diabetes risk

Around one in seven people between the age of 18 and 79 with selected citizenships in Germany without diagnosed diabetes have an elevated to very high risk of developing type 2 diabetes in the next five years. The mean risk in this sample is 0.9 %, i.e. on average, the respondents have an individual risk of around 1 in 100 people developing type 2 diabetes in the next five years. For the population in Germany as a whole, an average risk of 1.5 % and 1.1 % was calculated for the periods 1997 to 1999 (German National Health Interview and Examination Survey 1998, GNHIES98) and 2008 to 2011 (German Health Interview and Examination Survey for Adults, DEGS1), respectively [42]. Considering the decreasing risk over time observed in the earlier surveys, the calculated risk in the study at hand appears plausible overall. It should be noted that the sample of people without diabetes in the current study is, on average, younger than the participants in the earlier surveys in the general population (41.0 years; GNHIES98: 46.4 years; DEGS1: 46.0 years) [33] and may therefore also be at a slightly lower risk due to their age. Overall, the risk of developing type 2 diabetes in the next five years does not appear to be higher among people of the selected citizenships compared to the general population. As in GNHIES98 and DEGS1, the present study shows a higher risk of diabetes in men than in women, in the low compared to the middle and high education groups and with increasing age [33].

No differences were found in diabetes risk according to self-reported experiences of discrimination in everyday life, in the health or care sector or according to the sense of belonging to the society in Germany. However, a higher risk of diabetes was observed in people with lower German language proficiency. This could be due to an impeded access to prevention programmes and health information [7, 43, 44]. Lower German language proficiency might also be associated with a poorer socioeconomic situation due to disadvantages in terms of employment, housing and education, which is linked to a higher risk of diabetes [42, 45, 46].

#### Healthcare

Almost 90 % of 45- to 79-year-olds with selected citizenships and type 2 diabetes are treated with antidiabetic drugs. This roughly corresponds to the proportion of people with type 2 diabetes aged 45 and over who are treated pharmacologically in the nationwide study German Health Update (GEDA) 2021/2022-Diabetes [47]. In addition, comparable to the respondents from GEDA 2021/2022-Diabetes, around one third are treated with insulin (alone or in combination with other medications) and there are no sex-related differences.

The type of medication does not vary according to German language proficiency, self-reported experiences of discrimination in everyday life and in the health or care sector and according to the sense of belonging to the society in Germany. In contrast to GEDA 2021/2022-Diabetes, there are pronounced differences with regard to education, with a higher proportion of insulin treatment in the group with lower educational level. This might hint to a less favorable course of the disease in people with a history of migration in the low education group. According to the National Treatment Guideline, a potential indication for insulin therapy is only given if the individually determined therapy goal is not achieved despite treatment with non-pharmacological measures and antidiabetic drugs other than insulin [13].

Among people with selected citizenships, less than 60 % stated that they self-monitor their blood glucose, similar to the sample with type 2 diabetes from the general population based on GEDA 2021/2022-Diabetes. Given the recommendation for blood glucose self-monitoring for all types of diabetes [48], these results suggest a potential for improving self-management that applies to the entire population with type 2 diabetes. Further indicators on self-management could not be included in this study. Similar to those surveyed in GEDA 2021/2022-Diabetes, over 90 % of respondents in this study also reported that their blood glucose had been measured in the last 12 months as part of their received healthcare. However, unlike in GEDA 2021/2022-Diabetes, they were not asked directly if the HbA1c had been determined, so that the measurement of the blood glucose level may also have been considered. For self-monitoring and medical monitoring of blood glucose, there were no differences according to the analysed migration-related characteristics.

#### Complications

Almost one third of the 45- to 79-year-old respondents with type 2 diabetes from the present study report at least one of the diabetes-related complications considered (32.6 %), compared to around a quarter among people with type 2 diabetes aged 45 and older from the GEDA 2021/2022-Diabetes survey (26.7 %) [47].

In the present study, the proportion of at least one diabetes-related complication was higher among those with self-reported experiences of discrimination in the health or care sector. Where there is an increased risk of complications, a delay or absence of healthcare due to discrimination can lead to serious consequences [6, 49]. However, based on a cross-sectional design, the direction of the relationship between complications and discrimination cannot be determined. It is also conceivable that people with complications more frequently have contact with the healthcare system and hence, experience more discrimination in this context.

#### Concomitant diseases

While depressive symptoms are around twice as frequent in people with diabetes in the general population as in people without diabetes [50], in the present analysis, no significant difference can be observed in 45- to 79-year-olds with selected citizenships with and without diabetes. Recent studies, both in the general population [51] as well as in the overall GEDA Fokus sample [1], found a lower prevalence of depressive symptoms in older adulthood compared to middle age. In this study, the difference was observed accordingly for persons without diabetes (65 – 79 years: 10.0 % vs. 45 – 64 years: 15.7 %), while in persons with diabetes depressive symptoms were observed just as frequently in the older age group as in the younger age group (20.7 % vs. 20.4 %). In a qualitative study conducted in 2013 with predominantly over 65-year-olds with type 2 diabetes and a history of migration (from Bosnia to Sweden), half reported depressive symptoms and challenges (including language barriers) in dealing with the disease on a daily basis [52]. It is possible that older people with a history of migration, in particular, experience desperateness and helplessness in face of the diagnosis [24, 52] which can contribute to the development of depressive symptoms. However, further research is needed on this.

In the present study, in bivariate analyses depressive symptoms were observed more frequently in people with type 2 diabetes if they had a lower level of German language proficiency. These differences were not confirmed in the multivariable analysis. Further research based on larger samples is needed to investigate the relationship. There are indications that people with diabetes and depressive symptoms are significantly less likely to receive psychotherapeutic treatment in the case of language barriers [53].

In both people with and without diabetes, experiences of discrimination in everyday life and in the health or care sector are associated with depressive symptoms (cf. for the overall GEDA Fokus sample [12]). Literature hints to an association between discrimination with depressive symptoms and diabetes-related distress, which in turn can be linked to an unfavorable metabolic situation [54]. A strong sense of belonging to the society in Germany as a protective factor against depression [55] was only found in persons without diabetes. In this group, a strong compared to a weak sense of belonging to the society in Germany was associated with reporting depressive symptoms less often, while there were no such differences observed in persons with diabetes.

As expected, cardiovascular diseases were reported more than twice as often among people with diabetes compared to those without diabetes in the current study [56]. Overall, cardiovascular disease was observed in more than a third of people with diabetes. Older age (65 – 79 years) was associated with a higher prevalence of cardiovascular disease in both people with and without diabetes.

While there were no differences in this study for persons with diabetes with regard to cardiovascular disease stratified by migration-related variables, the results for persons without diabetes were more in line with expectations: Those with fewer experiences of discrimination in everyday life or in the health or care sector and a stronger sense of belonging to the society in Germany less often reported cardiovascular disease. Other studies indicate an association between experiences of discrimination and an increased risk of developing cardiovascular diseases and suffering acute events, including heart attacks and strokes, over the next ten years [9, 57].

As expected, hypertension is more than twice as frequent in people with type 2 diabetes than in people without diabetes in this study [58]; almost 70 % of people with type 2 diabetes reported high blood pressure. There are no age-related differences for persons with diabetes, while in persons without diabetes high blood pressure is less frequently observed at a younger age. An association between experiences of discrimination and high blood pressure described in the literature [59] is shown in the present study for persons without but not with diabetes regarding discrimination in the health or care sector. In bivariate analyses, among persons with type 2 diabetes with a stronger sense of belonging the to society in Germany, a higher proportion with high blood pressure was observed in this study. However, this is a borderline significant association, which is no longer evident when adjusting for sociodemographic characteristics and requires further research.

### 4.2 Strengths and limitations

The article is based on a large nationwide sample of people with selected citizenships, which represent some of the major migration groups in Germany. However, the results cannot be generalised for all people with a history of migration in Germany, as citizenship is the only characteristic according to which the sample was drawn, meaning that, for example, naturalised people with only German citizenship were not included. The response rate of 18,4 %is comparatively low, hence, a selection bias in the willingness to participate cannot be ruled out. However, utilising a comprehensive recruitment strategy offering different survey modes and study languages, accessibility to study participation and the response rate could be increased [60, 61].

In light of the heterogeneity in the living situations of people with a history of migration, it should be noted that only selected migration-related characteristics that appear particularly relevant and modifiable in the context of prevention and healthcare, including experiences of discrimination in the health or care sector, were considered. In the multivariable models, in addition to sex, age and education, we also controlled for citizenship according to residents’ registries. It should be noted that other migration-related characteristics such as duration of residence, residence status or country of birth could not be included in the present analysis due to low case numbers in relation with some diabetes indicators. A few components for determining the diabetes risk could not be collected directly in the study population. Therefore, they were calculated on the basis of available data using a regression equation in accordance with a procedure used in previous analyses (waist circumference [34]) or used as a constant on the basis of data from the general population (family history [33]). It is therefore possible that the results would have been different if all the data required by the GDRS had been available. Some results, particularly on medication, depressive symptoms and diabetes-related complications, are subject to greater uncertainty due to a relatively high proportion of missing values or small case numbers, which is reflected accordingly in the width of the 95 % CI. In addition, due to the relatively small number of cases, the categories of the migration-related characteristics had to be summarised (e.g. 5-point Likert scale categorised into two groups), which is associated with a loss of information. Due to the cross-sectional design, it is not possible to make any statements about the sequence of cause and effect of the associations described, e.g. experiences of discrimination in the health or care sector could lead to an avoidance of examinations and thus an increased risk of complications, on the other hand, complications could also lead to more frequent contact with the healthcare system, in which discrimination and stigmatization can occur.

This study provides an overview of a selection of different diabetes indicators by means of descriptive analyses according to different sociodemographic and migration-related characteristics. Future multivariable analyses, also including other migration-related characteristics such as duration of residence [12, 62] or the country of birth [63, 64] can provide deeper insights into the risk and healthcare situation of type 2 diabetes in people with selected citizenships. Not only should be investigated which associations exist independently of sex, age and education, but also whether interactions are observable that could not be considered in the present study.

### 4.3 Conclusion and practical implications

The risk of developing type 2 diabetes in the next five years does not appear to be increased overall among people with selected citizenships compared to the general population. The types of blood glucose-lowering medication are also distributed similarly to a sample of people with type 2 diabetes from the general population. Similarly, over 90 % of people with selected citizenships and type 2 diabetes reported receiving a blood glucose measurement from a healthcare professional within the last year. Comparable to a sample from the general population, only around two thirds of persons with type 2 diabetes report blood glucose self-monitoring, which indicates a general need to strengthen self-management.

In this article, we were able to show for the first time that, in addition to sociodemographic differences already known for the general population, there are also differences according to migration-related characteristics in association with type 2 diabetes within a sample of people with the selected citizenships. Low levels of German language proficiency and experiences of discrimination can be expected to result in health disadvantages in some areas, e.g. people with lower levels of German language proficiency are more likely to have a high risk of type 2 diabetes. There are also hints that people with diabetes are more likely to be affected by diabetes-related complications if they experience more frequent discrimination in the health or care sector and by depressive symptoms if they experience discrimination in everyday life or in the health or care sector. The results underline that in the prevention and healthcare of type 2 diabetes, particular attention should be paid to people who experience language barriers and discrimination.

Slightly less than a third of 18- to 79-year-olds without a diabetes diagnosis and slightly more than a third of 45- to 79-year-olds with type 2 diabetes in this study had moderate to very poor German language proficiency. This emphasises that health information on the prevention of diabetes should be accessible and understandable even with little or no knowledge of German. To increase knowledge about the interrelationship between diabetes and diet and exercise, communities and interest groups of people with a history of migration could be involved and information campaigns should be developed jointly [26]. There are already some multilingual materials available, e.g. checklists and infographics [65] as well as migration-sensitive diabetes training, but there are further structural barriers, e.g. in the billing of training courses and the provision and billing of language mediation [66].

Around 40 % of people with type 2 diabetes and selected citizenships report everyday discrimination and just under 20 % report discrimination in the health or care sector. Therefore, special attention should be paid to the prevention of discrimination in the healthcare sector, both on a direct interpersonal and structural level. Kajikhina et al (2023) concluded that health inequalities affecting people with a history of migration can be fuelled and reinforced by experiences of exclusion and (racial) discrimination [6]. This can have consequences for the course of the disease, quality of life and life expectancy, especially in the presence of a chronic disease such as type 2 diabetes. Training and sensitization of staff working in the healthcare sector, including students and trainees, as well as anti-racism training are necessary here [49, 67].

It should be emphasised that multilingual or more comprehensible information cannot completely eliminate inequalities in diabetes prevention and healthcare. Rather, there are structural barriers that can hinder health-promoting behaviour, the utilisation of preventive services and improved self-management of the disease, e.g. low financial resources for healthy nutrition and precarious working conditions [6, 68]. Therefore, an overall improvement in the conditions in which people live, learn, work and age is needed [69, 70].

## Key statements

- Among adults without diabetes, those with lower German language proficiency have a higher 5-year risk of type 2 diabetes.
- Among People with type 2 diabetes, the type of medication and proportion with blood glucose monitoring do not differ according to experiences of discrimination, sense of belonging to the society in Germany or German language proficiency.
- Diabetes-related complications are more common among those who have experienced discrimination in the health or care sector more often.
- Experiencing discrimination is associated with depressive symptoms, regardless of the presence of type 2 diabetes.

## Figures and Tables

**Table 1:** Analysed samples of persons with Croatian, Italian, Polish, Syrian or Turkish citizenship in Germany according to sociodemographic and migration-related characteristics. Source: GEDA Fokus

	Persons without diabetes, 18 – 79 years (n = 4,698); Analysis of the type 2 diabetes risk			Persons with type 2 diabetes, 45 – 79 years (n = 326); Analysis of healthcare, secondary and concomitant diseases			Persons without diabetes, 45 – 79 years (n = 2,018); Analysis of concomitant diseases (comparison group)		
	n*	%**	(95 % CI)**	n*	%**	(95 % CI)**	n*	%**	(95 % CI)**
**Gender**									
Women	2,349	46.4	(43.5 – 49.3)	140	41.6	(33.3 – 50.4)	1,032	48.5	(45.0 – 52.1)
Men	2,349	53.6	(50.7 – 56.5)	186	58.4	(49.6 – 66.7)	986	51.5	(47.9 – 55.0)
**Age**									
18 – 44 years	3,032	61.4	(58.3 – 64.4)						
45 – 64 years	1,346	31.4	(28.8 – 34.1)	182	53.6	(45.0 – 62.1)	1,601	80.5	(77.4 – 83.3)
65 – 79 years	320	7.3	(5.9 – 8.9)	144	46.4	(37.9 – 55.0)	417	19.5	(16.7 – 22.6)
**Education group**									
Low	981	33.8	(30.6 – 37.2)	157	59.7	(50.8 – 68.0)	625	47.3	(42.7 – 52.0)
Medium/high	3,704	66.2	(62.8 – 69.4)	163	40.3	(32.0 – 49.2)	1,377	52.7	(48.0 – 57.3)
**German language proficiency**									
Moderate/poor/very poor	1,373	26.4	(23.6 – 29.5)	137	40.5	(32.1 – 49.6)	652	30.1	(26.4 – 34.2)
Native language/very good/good	3,290	73.6	(70.5 – 76.4)	179	59.5	(50.4 – 67.9)	1,306	69.9	(65.8 – 73.6)
**Experiences of discrimination in everyday life**									
Very often/often/sometimes	1,892	40.2	(37.2 – 43.2)	122	43.0	(34.7 – 51.6)	642	34.3	(31.0 – 37.7)
Rarely/never	2,803	59.8	(56.8 – 62.8)	203	57.0	(48.4 – 65.3)	1,370	65.7	(62.3 – 69.0)
**Experiences of discrimination in the health or care sector**									
Very often/often/sometimes	619	13.3	(11.7 – 15.1)	46	19.8	(13.9 – 27.4)	213	11.2	(9.0 – 13.8)
Rarely/never	4,059	86.7	(84.9 – 88.3)	277	80.2	(72.6 – 86.1)	1,784	88.8	(86.2 – 91.0)
**Sense of belonging to the society in Germany**									
Partly/barely/not at all	1,813	35.5	(32.9 – 38.1)	110	36.1	(28.3 – 44.8)	656	32.9	(29.6 – 36.2)
Very strong/strong	2,841	64.5	(61.9 – 67.1)	214	63.9	(55.2 – 71.7)	1,338	67.1	(63.8 – 70.4)
**Total**	**4,698**	**100**		**326**	**100**		**2,018**	**100**	

* = unweighted,

** = weighted;

CI = confidence interval; education group according to CASMIN classification

Missing values: for persons without diabetes (18 – 79 years): n = 416 for type 2 diabetes risk, n = 13 for education group, n = 35 for German language proficiency, n = 3 for experiences of discrimination in everyday life, n = 20 for experiences of discrimination in the health or care sector, n = 44 for sense of belonging; for persons with type 2 diabetes (45 – 79 years): n = 6 for education group, n = 10 for German language proficiency, n = 1 for experiences of discrimination in everyday life, n = 3 for experiences of discrimination in the health or care sector, n = 2 for sense of belonging; for persons without diabetes (45 – 79 years): n = 16 for education group, n = 60 for German language proficiency, n = 6 for experiences of discrimination in everyday life, n = 21 for experiences of discrimination in the health or care sector, n = 24 for sense of belonging

**Annex Table 1:** Type 2 diabetes risk (proportions with 95 % confidence intervals) in persons with selected citizenships in Germany (18 – 79 years) according to sociodemographic characteristics (n = 2,349 women, n = 2,349 men). Source: GEDA Fokus

5-year risk of type 2 diabetes in categories	Low risk (< 2 %)			Still low risk (2 – < 5 %)			Elevated risk (5 – < 10 %)			High to very high risk (≥ 10 %)		
	n*	%**	(95 % CI)**	n*	%**	(95 % CI)**	n*	%**	(95 % CI)**	n*	%**	(95 % CI)**
**Gender**												
Women	1,912	78.7	(75.7 – 81.4)	229	10.8	(9.1 – 12.8)	114	5.4	(4,0 – 7.1)	94	5.1	(3.7 – 7.0)
Men	1,630	66.0	(62.2 – 69.6)	331	16.7	(14 – 19.8)	184	9.2	(7.3 – 11.5)	204	8.2	(6.6 – 10.1)
**Age**												
18 – 44 years	2,827	91.4	(89.4 – 93.1)	139	5.4	(4.1 – 7.0)	43	1.8	(1.1 – 3.0)	23	1.4	(0.8 – 2.5)
45 – 64 years	689	48.7	(44.1 – 53.4)	356	28.6	(24.8 – 32.7)	166	13.5	(10.7 – 16.9)	135	9.2	(7.2 – 11.6)
65 – 79 years	26	6.6	(3.8 – 11.1)	65	23.8	(16.7 – 32.7)	89	28.0	(21.3 – 35.9)	140	41.6	(34.2 – 49.4)
**Education group**												
Low	604	61.2	(56.1 – 66.0	173	20.0	(16.5 – 24.1)	88	8.7	(6.5 – 11.4)	116	10.2	(7.9 – 12.9)
Medium/high	2,930	77.4	(74.4 – 80.1)	385	10.9	(9.2 – 12.7)	209	6.7	(5.3 – 8.5)	180	5.0	(3.9 – 6.5)
**Total**	**3,542**	**71.9**	**(69.1 – 74.4)**	**560**	**14.0**	**(12.3 – 15.8)**	**298**	**7.4**	**(6.1 – 8.9)**	**298**	**6.8**	**(5.6 – 8.1)**

n* = unweighted,

%** = weighted;

CI = confidence interval; education group according to CASMIN classification

Missing values: n = 8 for education group

**Annex Table 2:** Blood glucose-lowering medication (proportions with 95 % confidence intervals) in people with selected citizenships in Germany (45 – 79 years) with type 2 diabetes according to sociodemographic and migration-related characteristics (n = 140 women, n = 186 men). Source: GEDA Fokus

	Insulin with and without other antidiabetics			Only tablets or other medication to be injected			No antidiabetics		
	n*	%**	(95 % CI)**	n*	%**	(95 % CI)**	n*	%**	(95 % CI)**
**Gender**									
Women	31	32.5	(17.6 – 52.1)	56	57.6	(39.7 – 73.7)	12	9.9	(3.5 – 24.7)
Men	40	25.3	(15.3 – 38.9)	97	66.7	(53.9 – 77.4)	10	8.0	(2.7 – 21.4)
**Age**									
45 – 64 years	37	21.4	(13.1 – 33.1)	87	64.5	(51.3 – 75.8)	16	14.1	(6.1 – 29.4)
65 – 79 years	34	37.4	(23.7 – 53.4)	66	60.9	(45.0 – 74.8)	6	1.7	(0.6 – 4.7)
**Education group**									
Low	43	35.1	(23.1 – 49.5)	73	54.2	(41.7 – 66.1)	13	10.7	(4.4 – 23.6)
Medium/high	28	15.5	(8.7 – 25.9)	78	79.7	(68.5 – 87.6)	8	4.9	(2.1 – 10.8)
**German language proficiency**									
Moderate/poor/very poor	30	31.2	(19.0 – 46.6)	69	60.9	(45.1 – 74.7)	8	8.0	(2.5 – 22.5)
Native language/very good/good	39	23.1	(13.9 – 36.0)	84	67.2	(55.9 – 76.8)	14	9.7	(4.0 – 21.5)
**Experiences of discrimination in everyday life**									
Very often/often/sometimes	22	22.2	(11.0 – 39.8)	60	64.6	(48.8 – 77.8)	11	13.1	(5.4 – 28.5)
Rarely/never	49	33.1	(23.2 – 44.9)	93	61.6	(49.8 – 72.2)	11	5.3	(2.0 – 13.0)
**Experiences of discrimination in the health or care sector**									
Very often/often/sometimes	15	44.7	(21.9 – 69.9)	20	50.9	(26.6 – 74.7)	1	4.5	(0.6 – 27.5)
Rarely/never	56	24.4	(16.9 – 34.0)	132	65.6	(56.2 – 74.0)	21	9.9	(4.6 – 20.3)
**Sense of belonging to the society in Germany**									
Partly/barely/not at all	24	21.4	(12.5 – 34.1)	44	61.6	(44.0 – 76.6)	11	17.1	(6.3 – 38.8)
Very strong/strong	46	31.7	(20.4 – 45.6)	108	63.7	(50.1 – 75.4)	11	4.6	(1.4 – 14.7)
**Total**	**71**	**28.3**	**(20.0 – 38.4)**	**153**	**62.9**	**(53.1 – 71.8)**	**22**	**8.8**	**(4.1 – 17.9)**

* = unweighted,

** = weighted;

CI = confidence interval; education group according to CASMIN classification

Missing values: n = 80 total, n = 3 for education group, n = 2 for German language proficiency, n = 1 for discrimination in the health or care sector, n = 2 for sense of belonging to the society in Germany

**Annex Table 3:** Blood glucose self-monitoring and blood glucose measurement by a healthcare professional (proportions with 95 % confidence intervals) in people with selected citizenships in Germany (45 – 79 years) with type 2 diabetes stratified by sociodemographic and migration-related characteristics (n = 140 women, n = 186 men). Source: GEDA Fokus

	Blood glucose self-monitoring			Blood glucose measurement by a healthcare professional		
	n*	%**	(95 % CI)**	n*	%**	(95 % CI)**
**Gender**						
Women	78	66.2	(53.3 – 77.0)	123	93.1	(84.3 – 97.1)
Men	117	51.2	(39.0 – 63.2)	173	93.0	(83.7 – 97.2)
**Age**						
45 – 64 years	115	60.1	(47.6 – 71.4)	165	90.2	(79.4 – 95.6)
65 – 79 years	80	54.2	(41.8 – 66.1)	131	96.4	(92.2 – 98.4)
**Education group**						
Low	93	59.1	(45.6 – 71.3)	144	93.1	(82.5 – 97.5)
Medium/high	100	55.3	(41.4 – 68.5)	148	92.9	(84.4 – 96.9)
**German language proficiency**						
Moderate/bad/very bad	84	62.0	(50.9 – 72.1)	126	93.4	(84.0 – 97.4)
Native language/very good/good	106	54.4	(40.8 – 67.3)	160	92.2	(82.7 – 96.7)
**Experiences of discrimination in everyday life**						
Very often/often/sometimes	73	58.2	(41.3 – 73.3)	109	90.1	(77.4 – 96.1)
Rarely/never	121	56.8	(46.0 – 66.9)	186	95.3	(89.3 – 98.0)
**Experiences of discrimination in the health or care sector**						
Very often/often/sometimes	33	68.1	(44.2 – 85.2)	41	95.1	(88.6 – 97.9)
Rarely/never	161	55.1	(44.7 – 65.0)	252	92.5	(84.8 – 96.4)
**Sense of belonging to the society in Germany**						
Partly/barely/not at all	66	56.1	(42.4 – 68.9)	100	89.9	(72.9 – 96.7)
Very strong/strong	128	58.2	(46.4 – 69.2)	194	94.8	(89.9 – 97.4)
**Total**	**195**	**57.4**	**(47.9 – 66.4)**	**296**	**93.0**	**(86.8 – 96.4)**

* = unweighted,

** = weighted;

CI = confidence interval; education group according to CASMIN classification

Missing values: n = 8 for blood glucose self-monitoring (additionally n = 3 for education group, n = 10 for German language proficiency, n = 1 for experiences of discrimination in everyday life, n = 3 for experiences of discrimination in the health or care sector, n = 2 for sense of belonging to thesociety in Germany), n = 6 for measurement of blood sugar by ahealth professional (additionally n = 4 for education group, n = 10 for German language proficiency, n = 1 for experiences of discrimination in everyday life, n = 3 for experiences of discrimination in the health or care sector, n = 2 for sense of belonging to the society in Germany)

**Annex Table 4:** Diabetes-related secondary diseases (proportions with 95 % confidence intervals) in persons with selected citizenships in Germany (45 – 79 years) with type 2 diabetes according to sociodemographic and migration-related characteristics (n = 140 women, n = 186 men). Source: GEDA Fokus

	Any diabetes-related complication		
	n*	%**	(95 % CI)**
**Gender**			
Women	38	41.7	(25.7 – 59.6)
Men	44	25.9	(16.1 – 38.9)
**Age**			
45 – 64 years	39	26.4	(15.9 – 40.5)
65 – 79 years	43	40.2	(25.1 – 57.4)
**Education group**			
Low	55	35.9	(23.5 – 50.5)
Medium/High	25	26.0	(13.3 – 44.4)
**German language proficiency**			
Moderate/bad/very bad	44	45.7	(29.7 – 62.7)
Native language/very good/good	35	24.5	(12.2 – 43.1)
**Experiences of discrimination in everyday life**			
Very often/often/sometimes	36	37.7	(22.8 – 55.4)
Never or rarely	46	28.6	(18.2 – 41.9)
**Experiences of discrimination in the health or care sector**			
Very often/often/sometimes	20	60.3	(38.2 – 78.9)
Rarely/never	62	26.2	(16.6 – 38.6)
**Sense of belonging to the society in Germany**			
Partly/barely/not at all	29	29.8	(17.0 – 46.8)
Very strong/strong	52	34.1	(20.3 – 51.1)
**Total**	**82**	**32.6**	**(22.2 – 45.0)**

* = unweighted,

** = weighted;

CI = confidence interval; education group according to CASMIN classification

Missing values: n = 51, additionally n = 4 for education, n = 8 for German language proficiency, n = 3 for experiences of discrimination in the health or care sector, n = 1 for sense of belonging to the society in Germany

**Annex Table 5:** Concomitant diseases (proportions with 95 % confidence intervals) in people of selected citizenships in Germany aged 45 – 79 years with type 2 diabetes (n = 140 women, n = 186 men) compared to people without diabetes (n = 1,032 women, n = 986 men) according to sociodemographic characteristics. Source: GEDA Fokus

	Depressive symptoms						Cardiovascular diseases						High blood pressure					
	Type 2 diabetes			No diabetes			Type 2 diabetes			No diabetes			Type 2 diabetes			No diabetes		
	n	%	(95 % CI)	n	%	(95 % CI)	n	%	(95 % CI)	n	%	(95 % CI)	n	%	(95 % CI)	n	%	(95 % CI)
**Gender**																		
Women	30	31.2	(21.1 – 43.5)	151	15.8	(12.3 – 20.1)	37	37.0	(22.9 – 53.8)	94	11.7	(8.7 – 15.7)	97	73.7	(62.5 – 82.6)	311	33.2	(27.9 – 39.0)
Men	33	13.0	(7.7 – 21.1)	138	13.4	(10.2 – 17.4)	70	39.8	(30.3 – 50.3)	150	17.5	(13.7 – 22.1)	121	64.8	(53.0 – 75.1)	301	29.2	(25.5 – 33.3)
**Age**																		
45 – 64 years	41	20.4	(13.1 – 30.5)	245	15.7	(12.5 – 19.4)	45	27.4	(18.6 – 38.3)	147	11.0	(8.4 – 14.1)	111	60.8	(48.4 – 71.9)	402	26.5	(23.1 – 30.3)
65 – 79 years	22	20.7	(11.8 – 33.6)	44	10.0	(6.6 – 15.0)	62	52.5	(38.0 – 66.6)	97	30.7	(23.8 – 38.7)	107	77.4	(65.1 – 86.2)	210	50.2	(42.1 – 58.3)
**Education group**																		
Low	33	23.8	(15.8 – 34.3)	96	14.3	(10.3 – 19.5)	56	42.0	(30.7 – 54.2)	110	17.0	(12.9 – 22.1)	101	65.6	(54.1 – 75.6)	206	32.0	(27.1 – 37.3)
Medium/high	28	15.6	(8.1 – 27.9)	191	14.9	(11.5 – 19.1)	47	33.1	(21.6 – 47.0)	131	12.6	(9.6 – 16.4)	111	72.1	(60.3 – 81.5)	402	30.6	(26.7 – 34.8)
**Total**	**63**	**20.5**	**(14.2 – 28.8)**	**289**	**14.6**	**(12.0 – 17.6)**	**107**	**38.7**	**(30.2 – 48.0)**	**244**	**14.7**	**(11.9 – 18.0)**	**218**	**68.5**	**(60.1 – 75.9)**	**612**	**31.2**	**(27.8 – 34.7)**

Missing values: n = 12 for depressive symptoms in people with type 2 diabetes, n = 55 for depressive symptoms in people without diabetes, n = 13 for cardiovascular disease in people with type 2 diabetes, n = 72 for cardiovascular disease in people without diabetes, n = 1 for high blood pressure in people with type 2 diabetes, n = 35 for high blood pressure in people without diabetes
